# Simultaneous integrated protection: A new concept for high-precision radiation therapy

**DOI:** 10.1007/s00066-016-1057-x

**Published:** 2016-10-18

**Authors:** Thomas B. Brunner, Ursula Nestle, Sonja Adebahr, Eleni Gkika, Rolf Wiehle, Dimos Baltas, Anca-Ligia Grosu

**Affiliations:** 1 Department of Radiation Oncology, University Hospitals Freiburg, Freiburg, Deutschland; 2 Partner Site Freiburg, German Cancer Consortium (DKTK), Heidelberg, Germany

**Keywords:** Stereotactic body radiation therapy, Intensity-modulated radiotherapy, Efficacy, Toxicity, Organs at risk, Stereotaktische Körperbestrahlung, Intensitätsmodulierte Strahlentherapie, Effizienz, Toxizität, Risikoorgane

## Abstract

**Objective:**

Stereotactic radiotherapy near serial organs at risk (OAR) requires special caution. A novel intensity-modulated radiotherapy (IMRT) prescription concept termed simultaneous integrated protection (SIP) for quantifiable and comparable dose prescription to targets very close to OAR is described.

**Materials and methods:**

An intersection volume of a planning risk volume (PRV) with the total planning target volume (PTV) defined the protection volume (PTV_SIP_). The remainder of the PTV represented the dominant PTV (PTV_dom_). Planning was performed using IMRT. Dose was prescribed to PTV_dom_ according to ICRU in 3, 5, 8, or 12 fractions. Constraints to OARs were expressed as absolute and as equieffective doses at 2 Gy (EQD2). Dose to the gross risk volume of an OAR was to respect constraints. Violation of constraints to OAR triggered a planning iteration at increased fractionation. Dose to PTV_SIP_ was required to be as high as possible within the constraints to avoid local relapse.

**Results:**

SIP was applied in 6 patients with OAR being large airways (*n* = 2) or bowel (*n* = 4) in 3, 5, 8, and 12 fractions in 1, 3, 1, and 1 patients, respectively. PTVs were 14.5–84.9 ml and PTV_SIP_ 1.8–3.9 ml (2.9–13.4 % of PTV). Safety of the plans was analyzed from the absolute dose–volume histogram (dose to ml). The steepness of dose fall-off could be determined by comparing the dose constraints to the PRVs with those to the OARs (Wilcoxon test *p* = 0.001). Constraints were respected for the corresponding OARs. All patients had local control at a median 9 month follow-up and toxicity was low.

**Conclusion:**

SIP results in a median dose of ≥100 % to PTV, to achieve high local control and low toxicity. Longer follow-up is required to verify results and a prospective clinical trial is currently testing this new approach in chest and abdomen stereotactic body radiotherapy.

**Electronic supplementary material:**

The online version of this article (doi: 10.1007/s00066-016-1057-x) contains supplementary material, which is available to authorized users.

## Zusammenfassung

**Zielsetzung:**

Die stereotaktische Radiotherapie nahe serieller Risikoorgane (OAR) erfordert besondere Vorsicht. Wir beschreiben ein neues Konzept für die intensitätsmodulierte Strahlentherapie (IMRT), genannt simultan integrierte Protektion (SIP), das die Dosisverschreibung auf Zielvolumina in unmittelbarer Nähe von OAR quantifiziert.

**Material und Methoden:**

Das Überschneidungsvolumen eines Planungsrisikovolumens (PRV) mit dem gesamten Planungszielvolumen (PTV) definierte das Protektionsvolumen (PTV_SIP_). Der Rest des PTV repräsentierte das dominierende PTV (PTV_dom_). Die Planung erfolgte mittels IMRT. Nach ICRU wurde die Dosis auf PTV_dom_ in 3, 5, 8 oder 12 Fraktionen verschrieben. OAR-Grenzdosen wurden als absolute und äquieffektive Dosen zu 2 Gy (EQD2) überprüft. Die Dosis auf das makroskopische Risikovolumen eines OAR musste die Constraints einhalten. Eine Verletzung der OAR-Constraints triggerte eine Planungswiederholung mit höherer Fraktionszahl. Die PTV_SIP-Dosis_ sollte so hoch wie möglich sein, aber innerhalb der Constraints, um Lokalrezidive zu vermeiden.

**Ergebnisse:**

Die SIP-Technik wurde bei 6 Patienten mit den OARs große Atemwege (*n* = 2) und Darm (*n* = 4) in 3, 5, 8 und 12 Fraktionen bei jeweils 1, 3, 1 und 1 Patienten angewendet. PTVs maßen 14,5–84,9 ml und PTV_SIP_ 1,8–3,9 ml (2,9–13,4 % des PTV). Die Verträglichkeit der Pläne wurde durch die Analyse der absoluten Dosisvolumenhistogramme (Dosis auf ml) überprüft. Die Steilheit des Dosisabfalls wurde durch den Vergleich der Dosis-Constraints mit der Dosis auf die PRVs gegenüber der Dosis auf die OARs abgelesen (Wilcoxon-Test *p* = 0,001). Die Constraints für die OARs wurden eingehalten. Alle Patienten zeigten eine Lokalkontrolle bei einem medianen 9‑monatigen Nachbeobachtungszeitraum und niedrige Toxizität.

**Schlussfolgerung:**

SIP ermöglichte eine mediane Dosis von ≥100 % auf das PTV und ergab eine hervorragende Lokalkontrolle bei niedriger Toxizität. Die Verifikation der Ergebnisse erfordert eine längere Nachbeobachtungszeit. Eine prospektive klinische Studie testet derzeit diesen neuen Ansatz für die thorakale und abdominelle Körperstereotaxie.

## Introduction

Over the past two decades stereotactic radiotherapy (SRT) has evolved to a powerful tool to control lesions especially in the brain, lungs, and liver [11, 12, 19, 21, 23]. However, reports of high-grade toxicities after stereotactic body radiotherapy (SBRT) of central lung tumors and of lesions near the bowel or stomach on the other hand demonstrated the difficulties to safely administer SBRT in these situations despite multimodal imaging, accurate motion management, intensity-modulated radiotherapy (IMRT), and image-guided radiotherapy (IGRT) [9, 22, 26].

The concept of SRT relies on avoiding organs at risk (OARs) through high spatial precision. Inherently, limitations of SRT and SBRT were encountered when the target lesions were too close to OARs and this is the clinical problem stipulating the development of the currently described novel concept of simultaneous integrated protection (SIP). For example, perforation of central airways, bronchial hemorrhage, perforations of the esophagus, stomach, or small bowel were observed [7, 14, 26]. Strategies to reduce the risk of high-grade toxicities often rely on the prescription of reduced total dose to the entire planning target volume (PTV). However, reduction of the total dose comes at the price of lowered local tumor control [8, 20]. Another strategy is to increase the number of fractions to exploit the differential radiosensitivity of tumor and OARs as defined by their α/β ratios [1, 3]. While this will help to overcome some of the limitations in more critical locations, there are still a significant number of cases where neither reduction of the total dose nor increasing the number of fractions within the limits of significant hypofractionation enables the application of an adequate radiation dose. The above mentioned clinical reports of high-grade and even fatal complications after SBRT illustrate the consequences of nonadherence to these rules [14, 26]. The tradition of prescribing SBRT not according to ICRU but to individually chosen isodoses (typically 60–80 %) aggravates the problem due to steep dose gradients which make even small setup errors highly risky. In summary, there is no standard approach to overcome such obstacles of the safe application of SBRT at this time.

When change of dose or fractionation is not sufficient, this problem is often addressed by individual dose reductions at the interface of target lesions with a critical organ at risk (OAR) at the discretion of the treating physician. However, in addition to the lack of data in the literature, such compromises derived from the fear of normal tissue complications may lead to the application of insufficient tumor doses and impair local control [20]. Furthermore, the lack of interobserver and interinstitutional comparability is a cardinal factor of inconsistencies, and it is a problem for prospective trials.

Our aim was therefore to develop a prescription method maximizing consistency of SBRT hypofractionation plans for targets near OARs [5] which deliberately and in a calculated way lowers the dose to a part of the PTV that is close to critical OARs. We present a method named simultaneous integrated protection (SIP) in analogy to the simultaneous integrated boost (SIB) intensity-modulated radiotherapy (IMRT) technique. It is based on the definition of a subvolume being the intersection of the PTV and the PRV of a critical OAR to which the highest possible dose respecting the dose constraints for this OAR is planned and delivered.

## Materials and methods

The stepwise procedure to define the SIP approach is summarized in Table 1, while a flowchart is shown in Supplemental Fig. 1. The SIP concept was required for SBRT treatment planning in case where there was an unacceptable high dose to an OAR, i. e., if there was overlap of the PTV with either the OAR or its expansions (IRV, PRV). This represented the key inclusion criterion for this report. Patients with an indication for SBRT where the SIP concept did not achieve adequate protection of the OAR at the highest planned fraction number, i. e., 12 fractions, were not eligible as described below.

**Fig. 1:**
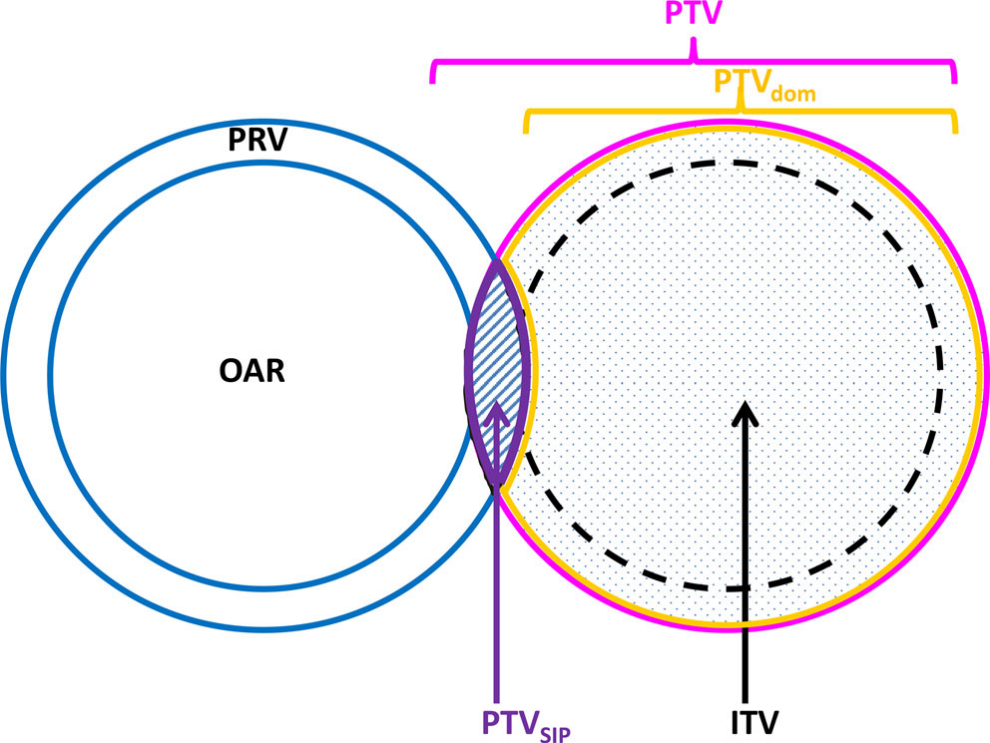
Contouring and planning using the simultaneous integrated protection (*SIP*) concept. Scheme of a critical organ at risk (*OAR*; *blue, left side*) with its planning risk volume (*PRV*) overlapping with the planning target volume (*PTV*, *pink*). The dominant PTV (PTV_dom_ = PTV\PRV; *orange*) is the prescribed dose in the conventional way, whereas the PTV_SIP_ (=PTV ∩ PRV; *purple*) is prescribed a lower dose to stay within the dose constraints for the OAR

This approach requires accurate definitions of the volumes to be treated. For the tumor, these were defined as GTV, CTV, ITV, and PTV in accordance with the ICRU reports 50 and 62. For the OAR, we used terms that are analogous with those for the tumors, i. e., OAR, IRV, and PRV. We defined the OAR as the volume segmented in the planning CT, and the IRV as the volume derived from 4D-CT. Describing the relation of PTV and the PRV to each other (Fig. 1), we defined the nomenclature of volumes in set theory notation to be 1) PTV for the total PTV, 2) the simultaneous protection volume (PTV_SIP_) for the intersection of the PTV and the PRV, PTV_SIP_ = PTV ∩ PRV, and 3) the PTV without intersection with the PRV as the dominant PTV, PTV_dom_ = PTV\PTV_SIP_. The term dominant was chosen to imply that the SIP approach is only valid for small volumes of PTV_SIP_.

Third, we defined the prescription of dose to the above volumes. The concept inherently requires an IMRT approach for simultaneous administration of different doses to PTV_dom_ and to PTV_SIP_. Dose was prescribed according to ICRU report 83 to PTV_dom_ indicating D_median_, D_98_, and D_02_. As typical for SBRT, we were asking for a table mount-like dose profile in PTV_dom_ and a D_02_ being up to 120 % of the prescribed dose [25]. In addition, the SIP concept was combined with a simultaneous integrated boost (SIB) in some of the patients. For PTV_SIP_, i. e., the volume that contains the dose gradient from PTV_dom_ to the OAR(s), the planning instructions were twofold:

- to stay within the boundaries of the given dose constraints for the OAR itself, and
- to make use of the maximum possible dose to PTV_SIP_ to minimize dose inhomogeneity for PTV.

In order to ensure this, the dose gradient between the dose to the OAR and the dose prescribed to the PTV_dom_ typically localizes to the PRV volume around the OAR. We also reported D_median_, D_98_, and D_02 _for PTV_SIP_ to quantify the dose sacrifice that was made for the PTV of a distinct lesion.

Fourth, we carefully chose available dose constraints for the OARs following the recommendations published by QUANTEC and other published recommendations commonly used for SBRT [1, 5, 12, 16, 27, 28]. For the respective fractionation schedules, dose constraints for OAR were calculated as equieffective doses in 2 Gy fractions (EQD2) with corresponding α/β ratios (α/β 3 for large airways, bowel structures).

**Fig. 2:**
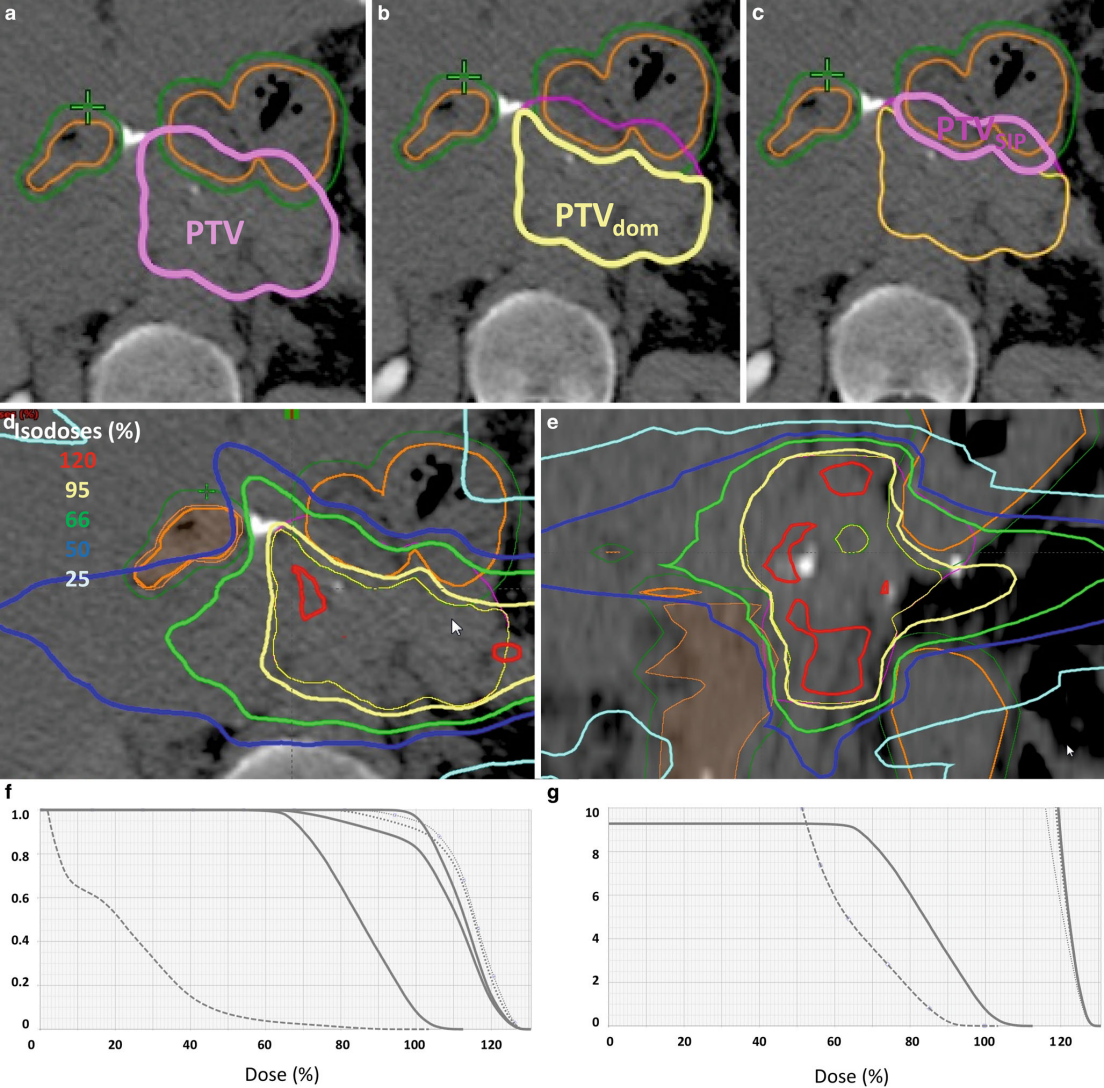
**a** The planning target volume (*PTV*, (*light pink*) intersects with planning risk volume (*PRV*, *green*) and organs at risk (*OAR*), small bowel (*orange*), in a patient with recurrent pancreatic cancer after resection. **b** The PRV is subtracted from PTV to define PTV_dom_ (*yellow*). **c** The PTV_SIP_ is defined as the intersecting region of PTV with the PRV (PTV ∩ PRV; *magenta*). **d,e** An intensity-modulated radiotherapy (*IMRT*) plan is developed to deliver full dose according to ICRU to PTV_dom_ and a lower dose to PTV_SIP_ respecting the dose constraints for small bowel in 12 fractions (D_max_ = 47.4 Gy, D_0.5ml_ = 44.5 Gy, D_5ml_ = 44.4 Gy). Isodose levels as stated on the *left side*. **f** Relative dose–volume histogram (*DVH*). Protection of the 9.2 ml PTV_SIP_ (*left solid*) compared with PTV (*middle solid*), ITV (*bold dotted*) and with PTV_dom _(*right solid*); gut (*dashes*). **g** Absolute DVH respecting the constraints for gut

Fifth, plans were checked for all boundaries as defined above prior to individual RTQA (Fig. 2). In cases where the dose constraints were violated at a chosen number of fractions, planning iterations with a higher number of fractions up to a specified maximum of 12 fractions were performed. Prescribed doses and dose constraints were recalculated to the EQD2 using the respective α/β ratios of tumor and OAR, and aiming to deliver iso-effective doses to the tumor with lower toxicity by protracted dose delivery. If the normal tissue constraints could not be fulfilled by increasing the number of fractions to the maximum number, SBRT was not given but conventionally fractionated treatment performed instead. Sixth, we excluded lesions with large absolute PTV_SIP_ volumes, with very small PTV as well as for single fraction radiosurgery from the use of the SIP concept. Seventh, we required high-precision patient positioning, motion management, and IGRT for the use of the SIP concept.

For the analysis descriptive statistics were used and the Wilcoxon test for paired differences was used for the comparison of dose to the PRVs with dose to the respective OARs to evaluate the plans for given dose constraints. Kaplan–Meier statistics were employed to calculate local control after therapy.

## Results

**Fig. 3:**
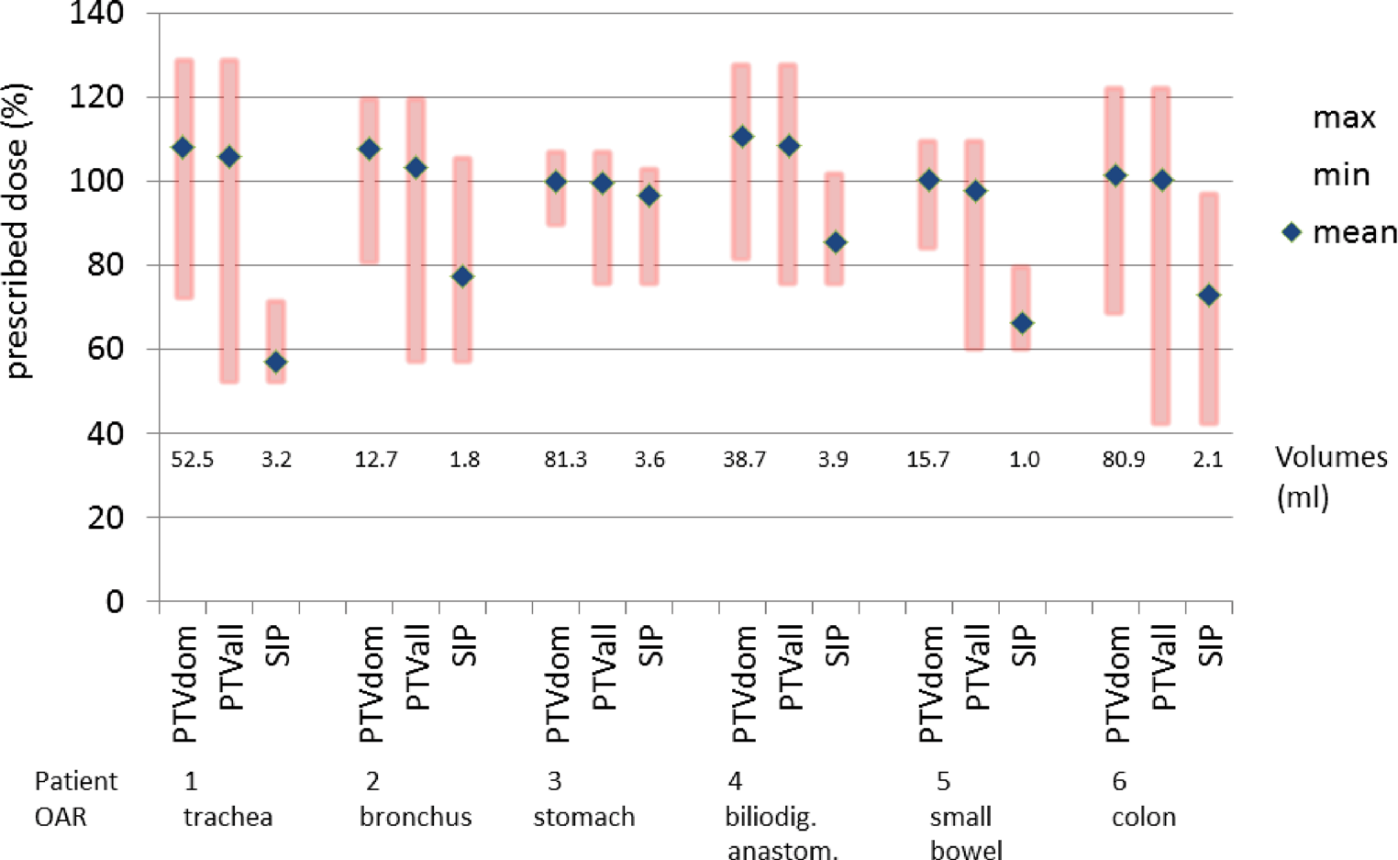
Dose to planning target volumes (*PTV*) in 6 patients planned with the simultaneous integrated protection (*SIP*) concept. For each patient from* left to right* the relative prescription doses to the dominant non-protection PTV , PTV, and PTV_SIP_ are shown with minimal (*min*), maximal (*max*), and mean (*filled diamonds*) relative doses. *OAR* organ at risk

In this article, we describe the clinical application of the SIP concept. Six patients with indications for SBRT of targets close to OAR underwent 4D treatment planning imaging with high-precision positioning. Two had lesions in the chest, one in the liver, two in the pancreas and one in the left kidney. One patient (# 3) was treated with a non-SIP SBRT plan with reduced dose (5 fractions of 6 Gy to 60 % isodose of ITV) for a central lung metastasis close to the right hilum but had an in silico SIP plan to full dose for PTV_dom_. All other patients were treated with the SIP plan. The size of the PTVs (PTV) ranged from 14.5–84.9 ml (median 49.15 ml, mean 49.57 ml; Fig. 3). Sizes of PTV protection subvolumes (PTV_SIP_) ranged from 1.0–3.9 ml (median 2.65 and mean 2.60 ml). Relative PTV_SIP_ ranged from 2.9–13.4 % of the size of PTV (median 5.9 %). Noteworthy, the largest ratio, 13.4 %, was an absolute volume of 2 ml, only. D_min_ of the PTV_SIP_ tended to be lower in patients 1, 2, and 6 due to air within the PTV_SIP_ volumes compared with the other patients. Safety of the plans was analyzed from the absolute volume DVHs as summarized in Supplemental Table 1 showing the comparison of the dose constraints with the doses in the plans to the OARs of the OARs and to the PRVs. The steepness of dose fall off can be read off by the comparing the doses to the PRVs with those to the OARs. Expectedly, the dose constraints for the respective OARs in the PRV volumes were violated for some of the PRVs but the constraints were respected for the corresponding OARs as an indirect sign for the successful application of the SIP concept (Supplemental Table 1). To quantify this, a comparison of the given dose constraints with the actual doses to the OAR volumes and the PRV volumes was performed by analyzing the difference of the ratio D (OAR)/D (constraint) with D (PRV)/D (constraint) for the 19 dose constraint values shown in Supplemental Table 1 (*p* = 0.001, Wilcoxon test). None of these patients showed severe toxicity within a median follow-up of 8.6 months (range 3.1–26.2 months) with favorable local control (100 %).

**Table 1:** Standardized definition of the simultaneous integrated protection (SIP) concept in the treatment planning algorithm

1. Indication: The use of the technique is indicated when	– First, the standard indications for stereotactic radiotherapy are given (not further specified in the framework of this manuscript) and	
	– Second, there is overlap of the planning target volume (PTV) with a critical organ at risk (OAR) or with the planning risk volume (PRV) of this OAR	
2. Definitions of volumes (Fig. 1):	– For the tumor: standard volumes GTV, CTV, ITV, and PTV are used (ICRU 50, 62)	
	– For the OAR: gross risk volume (OAR), planning risk volume (PRV) is used;	
	– For OARs with significant motion an internal risk volume (IRV) may be used to define the PRV	
	– Nomenclature of volumes (expressed in set theory notation):	– Total PTV: PTV
		– Simultaneous protection volume: PTV_SIP_ = PTV ∩ PRV
		– Dominant PTV: PTV_dom_ = PTV \ PTV_SIP_
3. Prescription:	– By definition the SIP concept is an application of IMRT	
	– Dose is prescribed according to ICRU (report 83) to PTV_dom_ reporting D_median_, D_98_, and D_02_	
	– D_median_ in PTV_dom_ should have a table mount-like dose profile as typical for stereotactic radiotherapy	
	– Within PTV_dom_ a classical simultaneous integrated boost volume (SIB) may be planned after definition of a respective SIB volume	
	– For PTV_SIP_, the transition volume from PTV_dom_ to OAR, the planning instructions are twofold:	– (1) Stay within the boundaries of the given dose constraints for the OAR
		– (2) Within (1), make use of the maximum possible dose to the OAR to minimise dose inhomogeneity for PTV
		– Report D_median_, D_98_, and D_02_
4. Dose constraints for OARs: dose constraints as published by QUANTEC or other are employed in biologically equivalent form, e. g., as EQD2 calculated with the appropriate α/β values. These need to be at the highest level of evidence available and have to be updated accordingly		
5. Analysis:	– Individual RTQA is performed if all boundaries are met at plan analysis for the PTV_dom_ and for the OAR	
	– If constraints are violated:	– Planning iterations with a higher number of fractions up to a specified maximum (e. g., 12 fractions) are performed
		– Here, prescribed doses and dose constraints are recalculated to the equieffective dose at 2 Gy (EQD2) using the respective α/β values of tumor and OAR aiming to deliver the maximum possible EQD2 to the tumor with acceptable toxicity
		– If the limits of the normal tissue constraints cannot be kept hereby, SBRT should not be given but rather conventionally fractionated treatment
6. IGRT: cutting edge patient positioning and IGRT is mandatory for the use of the SIP concept		

**Fig. 4:**
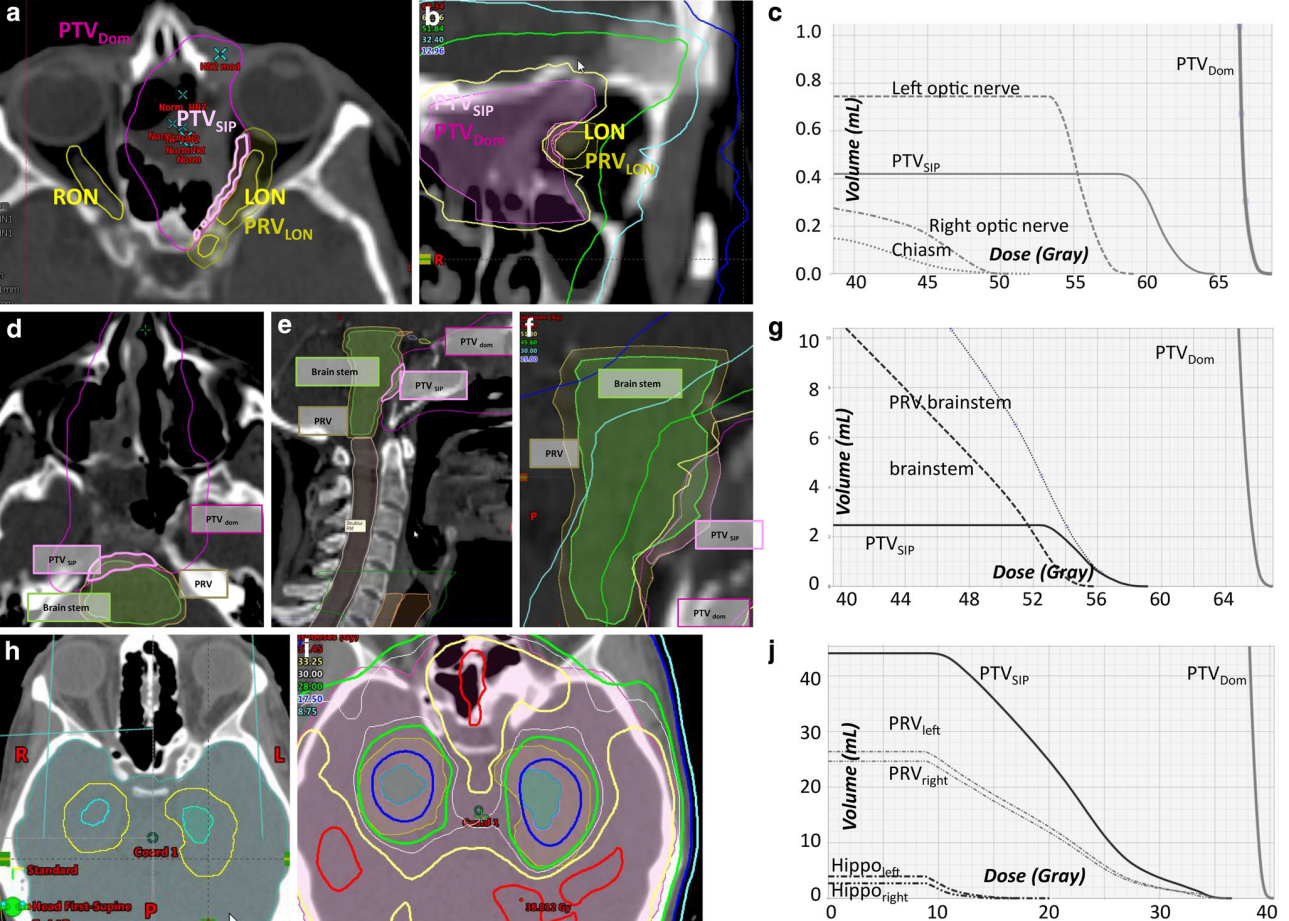
Examples for a simultaneous integrated protection (*SIP*) for the optic nerve, the brainstem, and the brachial plexus with dose parameters in Supplementary Table 2. **a** Axial planning CT of a patient with sinonasal squamous cell carcinoma who was treated with chemoradiotherapy after positive margin resection at the left optic nerve. As she refused left orbital exenteration, PTV_dom_ is treated with 64.8 Gy in 36 fractions. A 0.4 ml SIP volume is employed to respect a 60 Gy constraint to the left optic nerve. **b** The coronal plane visualizes the *yellow* 61.5 Gy isodose line around the nerve. **c** Absolute dose–volume histogram (*DVH*). **d** Axial and **e** sagittal planning CT of a patient with undifferentiated main nasal cavity carcinoma with initially direct contact to the brainstem which was shifted dorsally. Tumor shrinkage after two courses of induction chemotherapy with paclitaxel/cisplatin, then chemoradiotherapy with SIP-IMRT to 54 Gy during phase 1 followed by an adaptive sequential boost (not shown) during phase 2. **f** Isodoses at the interface between the PTV and the brainstem. **g** The brainstem constraint of 53 Gy is met as shown in the absolute DVH. **h–j** Hippocampus protection. **h** Delineation of the right (*sky blue*) and left (*blue-green*) hippocampus with the respective PRVs (*yellow*) that are generated by a 7 mm isotropic margin to the hippocampi. PTV_SIP_ corresponds to the PRV of the hippocampus minus the hippocampus itself (PTV_SIP_ = PRV_[side]_\hippocampus_[side]_). **i** A total dose of 35 Gy in 14 fractions was prescribed to the PTV_dom_. Note the 28.0 Gy (*green*) and the 17.5 Gy (*cornflower blue*) isodoses at the two SIP volumes. **j** In the absolute DVH, the hippocampi receive a mean dose of ≤10 Gy. *PRV* planning risk volume, *PTV* planning target volume

On the other hand, the dose trade-off to the PTVs due to SIP was also quantified. Mean doses to the PTVs were compared between the three volumes (PTV, PTV_dom_, PTV_SIP_) as shown in Table 2. Comparing D_mean, PTV_ with D_mean, _PTV_dom_, the difference was just about significant at *p* = 0.043 whereas the difference was more significant between D_mean, PTV_ with D_mean, _PTV_SIP_ at *p* = 0.028 (Wilcoxon test). Mean and median relative doses to 95 % (D95 vol.-%) of the volumes PTV_dom_, PTV, and PTV_SIP_ were 122, 105, and 90 %, as well as 120, 107, and 93 %, respectively. This reflects that the dose sacrifice to PTV_SIP_ was kept to a minimum. Maximum BED (α/β 10) doses in the PTV for the 6 patients were 124, 135, 93, 92, 114, and 154 Gy, respectively. Fig. 4 and Supplemental Table 2 show further examples of applications of the SIP concept for conventionally fractionated IMRT for cerebral and extracerebral target volumes.

**Table 2:** Relative dose parameters for all planning target volume (*PTV*) types in 6 patients

Patient	Target lesion	PTV type	D_mean_	D_min_	D_max_	D_98_ (%)	D_95_ (%)	D_02_ (%)	V_95 _(%)	V_107 _(%)
1	M_pul (CRC)	PTV	106.0	52.7	128.3	66.7	77.1	125.3	81.8	54.68
		PTV_dom_	108.19	72.7	128.3	85.9	89.8	125.4	86.96	58.09
		PTV_SIP_	70	52.7	86.5	56.3	58.5	81.2	0	0
2	NSCLC	PTV	110.2	60.7	125.4	70.2	73.3	122.8	87.4	79.1
		PTV_dom_	115.2	87.1	125.4	101.8	105.7	123	99.8	92.7
		PTV_SIP_	79.5	61.6	110.4	67.7	68.9	100.6	8.7	0.03
3	HCC	PTV	99.5	67.3	106.1	89.7	95.8	103.8	95.96	0
		PTV_dom_	100	90	106.1	96.5	97.8	102.7	99.4	0
		PTV_SIP_	67.3	75.9	102.3	85.5	88.9	101.0	75.56	0
4	LR-PDAC	PTV	108.4	75.9	126.9	94.1	96.5	124.7	97.02	60.65
		PTV_dom_	110.8	81.7	126.9	97.5	101.6	124.8	99.18	67.02
		PTV_SIP_	85.5	75.9	101.2	81.9	85.8	94.6	88.06	0
5	LR-PDAC	PTV	97.8	60.4	108.9	65.1	69.3	105.0	84.07	0.11
		PTV_dom_	100.5	84.4	108.9	92.7	93.4	105.1	93.33	0.12
		PTV_SIP_	66.4	60.4	78.6	62.1	62.6	74.8	0	0
6	Renal cancer	PTV	120.6	51.5	145.6	91.5	94.8	143.0	94.85	78.16
		PTV_dom_	121.6	82.7	145.6	93.4	96.3	143.1	96.43	80.35
		PTV_SIP_	87.7	51.5	115.1	57.2	61.0	109.3	39.6	5.04

Maximal doses were prescribed either with a table-mount profile (patients 1, 2, 4, 6) or without (patients 3, 5)

*HCC* hepatocellular carcinoma, *LR-PDAC* locally relapsed pancreatic cancer, *M_pul (CRC)* lung metastasis from colorectal cancer, *NSCLC* non-small cell lung cancer

## Discussion

The described technique of the SIP concept proposes a fully quantified method to protect OARs and to avoid toxicity in a deliberate and reproducible way, while keeping the dose to the remaining PTV at effective levels. The main advantage of this approach is the high level of transparency which makes it a suitable tool for multicenter trials in SBRT minimizing interinstitutional technical differences as a source of error. However, the concept is not restricted to SBRT but could also be used for conventional IMRT or even brachytherapy. In contrast to the SIB method where a small subvolume inside a PTV is prescribed to receive an escalated dose to enhance local control, the SIP concept prescribes a lower dose to a subvolume of a PTV with a high risk of severe toxicity.

The SIP concept is proposed for serial OARs according to the model of functional subunits (FSU) [29]. For serial organs, e. g., spinal cord, esophagus, and bowel, the defect of a few FSUs has a high likelihood for toxicity compared to parallel OARs such as the lung or the liver.

Using the SIP concept, the protection of an OAR is achieved by a protective outer shell around an OAR as a volume for the steep dose gradient between the tumor and the OAR. It is important to be aware that the definition of the protection volume of an OAR depends critically on its nature, e. g., PTV_SIP_ can be smaller to protect the chiasm compared to the stomach due to motion. For lesions in the chest, respiratory movements of OARs are of specific importance. Correspondingly, peristalsis of the gut is important in the abdomen [24]. Oral contrast prior to each fraction is recommended in upper abdominal SBRT for IGRT to visualize day-to-day changes. Summarizing, IGRT is key to verify whether employed margins of OARs are correct and clinical trials will have to verify whether the concept is useful and whether the dose constraints were correct. Adaptive radiotherapy strategies can also be combined with the SIP concept and we are currently analyzing this approach in prostate cancer IMRT for the rectum. However, we felt that this would be too complex in the framework of this article and therefore we plan to describe this in a subsequent publication.

Excessive contact volumes between the tumor and the OAR are not thought to be a good indication for SIP. It is not clear how large the PTV_SIP_ volume can be in absolute and relative values without a significant loss of tumor control. However, reporting D_median_, D_98_, and D_02_ for all target volumes can help to recognize the limits of SIP. Meticulous DVH analysis for target volumes and OARs alike is necessary, but currently, we cannot quantify the risk of local recurrence with SIP. Therefore, prospective trials have to evaluate local relapse and toxicity. The bystander effect may support local tumor control [18]. But it is advisable to exhaust the dose constraints to avoid the risk of local relapse. At this time it is not fully clear yet which dose parameters are most important for local tumor control, PTV encompassing dose, or maximum dose [15]. In a recent analysis of more than 1500 SBRT treatments of primary and secondary tumors of the lung with a broad range of primaries, a plateau of the dose–response curve with 90 % local tumor control probability was reached at 160 Gy BED when using the PTV maximum dose [13]. Their report supports the view to aim to a give a high dose to large parts of the PTV with a maximum dose at the center of the PTV where the likelihood of tumor location of a moving target is highest. If this concept is correct, then it might be possible to use the SIP concept with a lower dose in a peripheral subvolume of the PTV with lower likelihood of tumor cells being present without compromising local control. In this context it is also intriguing that in parotid sparing head and neck IMRT locoregional recurrences were not observed to occur predominantly in the spared areas but within the high-dose regions [1, 25]. The safety of SIP critically depends on the reliability of the chosen dose constraints which also need to be validated in prospective trials.

From the point of view of radiation biology, it should be stressed that the tumor front might harbor especially radioresistant subvolumes of the tumor. Such a pattern was described in rectal cancer after neoadjuvant therapy and resection [4, 10]. Epithelial mesenchymal transition (EMT) was described to be more prevalent in residual tumor subvolumes at the invasion front [4] which in turn was described to be enriched for cancer stem cells [2]. Another important factor of resistance to radiotherapy is hypoxia which is not restricted to the tumor core but also is found in subvolumes of the invasive front again warning from low doses at the edge of the tumor [6]. At the moment we cannot adequately image regions of hypoxia, stemness, and EMT in patients and therefore the dose sacrifices should always be as small as possible and this is a hallmark of the here described SIP technique.

In cases where the dose constraints are violated by very hypofractionated approaches (e. g., 3 or 5 fractions), more fractions can reduce the EQD2 for late toxicity due to low α/β values. Therefore, we use SIP up to ≤12 fractions for targets with intensive contact to OARs [17]. With the EQD2 formula, isoeffective and isotoxic fractionations should be calculated.

The prescription technique for SBRT described here allows accurate quantification of the dose delivered to dose limiting OARs based on the SIP approach. This system has two advantages: The dose sacrifice to the PTV due to the proximity to a dose-limiting OAR is fully quantified and can be used for local control analysis. At the same time the dose delivered to OARs and to PRVs of OARs can likewise be accurately quantified and therefore be used for toxicity evaluations. This method can be used for SBRT with all SBRT equipment and is suitable for multicenter trials.

## Conclusion

We present a concept for SBRT and IMRT close to high-risk OARs that is expected to be safe and effective and at the same time suitable for multicenter clinical testing. We currently test this approach in a single center phase I trial in patients with thoracic and abdominal lesions and we are confident to thereby further increase the safety of SBRT.

## Caption Electronic Supplementary Material

**Supplementary Figure 1** Flow chart for the making of a SIP plan.

**Supplementary Table 1** Dose constraints and dose parameters for the critical organs at risk and the planning risk volume.

**Supplementary Table 2** Dosimetric specifics for the treatment plans shown in Figure 4.
