# Supplementary material for: Simultaneous integrated protection: A new concept for high-precision radiation therapy
Source: Strahlenther Onkol. 2016 Oct 18;192(12):886–94. doi: 10.1007/s00066-016-1057-x (PMC5122615; doi:10.1007/s00066-016-1057-x)
Supplement: Supplementary file 1 — Supplementary Figure 1 Flow chart for the making of a SIP plan. [file 66_2016_1057_MOESM1_ESM.pptx]

## Slide 1
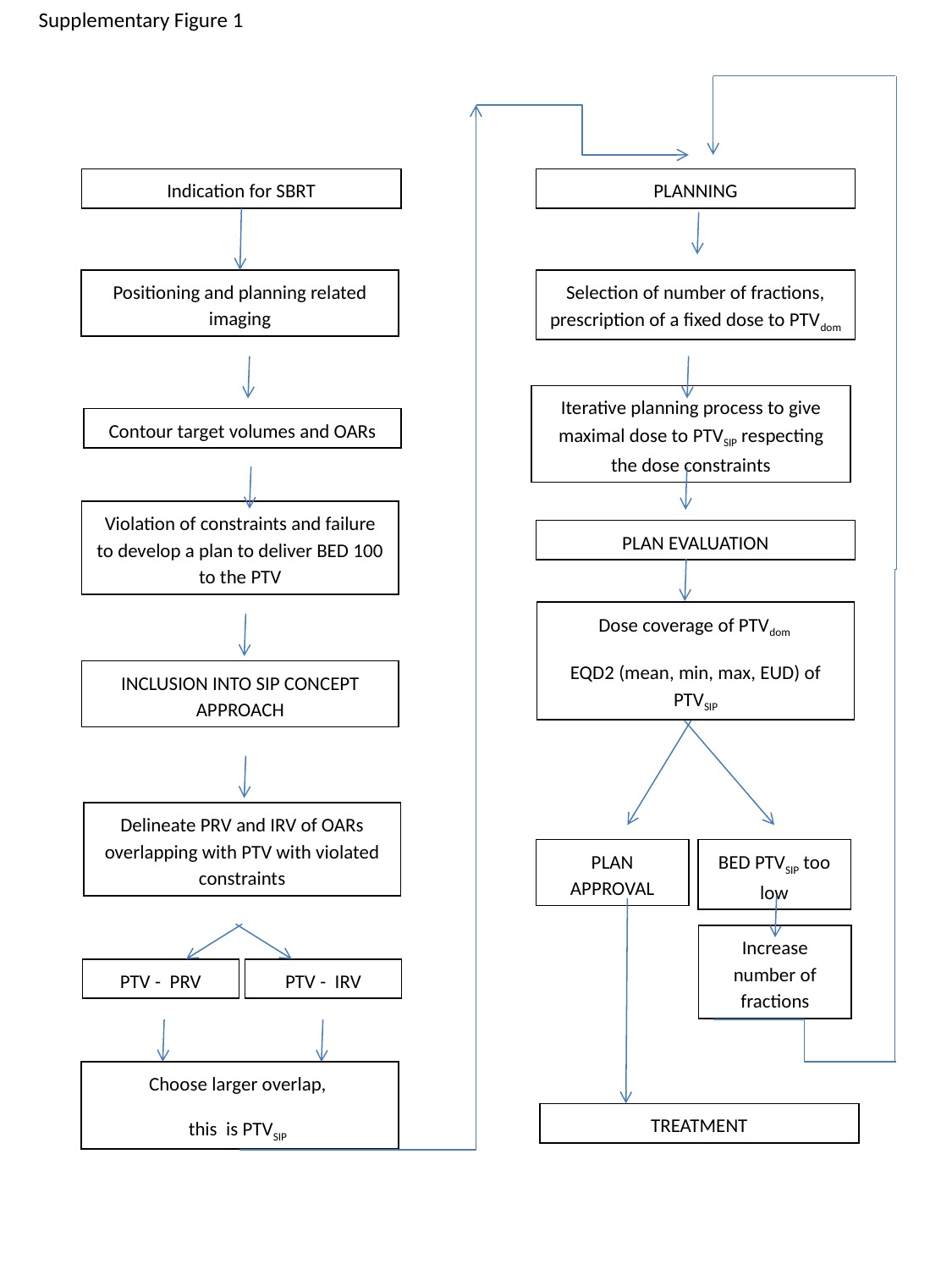

Supplementary Figure 1
Indication for SBRT
Planning
Positioning and planning related imaging
Selection of number of fractions, prescription of a fixed dose to PTVdom
Iterative planning process to give maximal dose to PTVSIP respecting the dose constraints
Contour target volumes and OARs
Violation of constraints and failure to develop a plan to deliver BED 100 to the PTV
Plan evaluation
Dose coverage of PTVdom
EQD2 (mean, min, max, EUD) of PTVSIP
INCLUSION INTO SIP CONCEPT APPROACH
Delineate PRV and IRV of OARs overlapping with PTV with violated constraints
Plan Approval
BED PTVSIP too low
Increase number of fractions
PTV - IRV
PTV - PRV
Choose larger overlap,
this is PTVSIP
Treatment
