# Supplementary material for: Simultaneous integrated protection: A new concept for high-precision radiation therapy
Source: Strahlenther Onkol. 2016 Oct 18;192(12):886–94. doi: 10.1007/s00066-016-1057-x (PMC5122615; doi:10.1007/s00066-016-1057-x)
Supplement: Supplementary file 2 — Supplementary Table 1 Dose constraints and dose parameters for the critical organs at risk and the planning risk volume. [file 66_2016_1057_MOESM2_ESM.docx]

**Supplementary Table 1.** Dose constraints and dose parameters for the critical organs at risk and the planning risk volume.

| **Patient**  **OAR** | **PTV_dom**  **prescription (Gy)** | **Dose parameters** |  | **Dose constraints for OARs** | | | **Actual doses to OARs^1^** | | | **Actual doses to PRVs** | | |
| --- | --- | --- | --- | --- | --- | --- | --- | --- | --- | --- | --- | --- |
|  |  |  | **#** | **SD**  **(Gy)** | **TD**  **(Gy)** | **EQD2**  **Gy_3_** | **SD (Gy)** | **TD (Gy)** | **EQD2**  **Gy_3_** | **SD**  **(Gy)** | **TD**  **(Gy)** | **EQD2**  **Gy_3_** |
| Patient 1 | 5 x 11 |  |  |  |  |  |  |  |  |  |  |  |
| Trachea |  | Dmax | 5 | 7.56 | 37.80 | 79.83 | 7.79 | 38.96 | 84.09 | 9.39 | 46.96 | 116.39 |
|  |  | D_0.5 | 5 | 7.26 | 36.30 | 74.49 | 6.98 | 34.89 | 69.63 | 8.37 | 41.83 | 95.09 |
| Patient 2 | 8 x 7.5 | Dmax | 8 | 5.72 | 45.76 | 79.81 | 5.76 | 46.11 | 80.82 | 7.85 | 62.81 | 136.31 |
| Prox bronchus |  | D_0.5 | 8 | 5.50 | 44.00 | 74.80 | 5.31 | 42.44 | 70.49 | 6.80 | 54.42 | 106.69 |
| Patient 3 |  | Dmax | 12 | 3.95 | 47.40 | 65.89 | 3.38 | 40.52 | 51.68 | 4.10 | 49.14 | 69.73 |
| Stomach |  | D_0.5 | 12 | 3.75 | 45.00 | 60.75 | 2.66 | 31.93 | 36.15 | 3.06 | 36.66 | 44.40 |
|  |  | D_5 | 12 | 3.70 | 44.40 | 59.50 | 1.94 | 23.26 | 22.97 | 1.79 | 21.46 | 20.55 |
|  |  | D_10 | 12 | 3.60 | 43.20 | 57.02 | 1.67 | 20.08 | 18.77 | 1.41 | 16.92 | 14.92 |
|  |  | D_15 | 12 | 3.50 | 42.00 | 54.60 | 1.51 | 18.12 | 16.34 | 1.20 | 14.37 | 12.06 |
| Patient 4 | 12 x 4 | Dmax | 12 | 3.95 | 47.40 | 65.89 | 3.50 | 42.03 | 54.66 | 4.26 | 51.13 | 74.25 |
| Small bowel |  | D_0.5 | 12 | 3.75 | 45.00 | 60.75 | 3.31 | 39.66 | 50.01 | 3.76 | 45.13 | 61.02 |
| BDA² |  | D_5 | 12 | 3.70 | 44.40 | 59.50 | 2.60 | 31.14 | 34.85 | 2.27 | 27.25 | 28.73 |
|  |  | D_10 | 12 | 3.60 | 43.20 | 57.02 | 1.95 | 23.36 | 23.11 | 1.73 | 20.74 | 19.61 |
| Patient 5 | 5 x 10 | Dmax | 5 | 6.75 | 33.75 | 65.81 | 6.80 | 34.02 | 66.71 | 8.56 | 42.82 | 99.03 |
| Small bowel |  | D_0.5 | 5 | 6.40 | 32.00 | 60.16 | 6.10 | 30.48 | 55.45 | 6.94 | 34.72 | 69.05 |
|  |  | D_5 | 5 | 6.30 | 31.50 | 58.59 | 4.42 | 22.11 | 32.82 | 5.60 | 28.01 | 48.19 |
|  |  | D_10 | 5 | 6.20 | 31.00 | 57.04 | 4.00 | 20.01 | 28.02 | 4.90 | 24.52 | 38.76 |
| Patient 6 | 3 x 15 | Dmax³ | 3 | n.a. | n.a. | n.a. | 11.04 | 33.11 | 92.94 | 18.20 | 54.59 | 231.50 |
| Colon |  | D3 | 3 | 13.3 | 39.90 | 130.07 | 7.31 | 21.92 | 45.18 | 9.68 | 29.03 | 73.60 |
|  |  | D20 | 3 | 6.50 | 19.50 | 37.05 | 4.53 | 13.59 | 20.47 | 5.39 | 16.18 | 27.16 |

Dose was given in equivalent dose in 2 Gy fractions (EQD2) at the respective α/β ratio for the organ at risk of interest (in this case 6 Gy). EDQ2 = D * {[d + (α/β)]/[2 Gy + (α/β)]}, where D is the total dose, d is the single dose, and α/β is the assumed ratio in Gray for a late side effect of an organ at risk. For each, constraints, OAR and PRV the dose was given as single dose (SD) and total dose (TD) for the numbers of fractions used for the specific patient (#). Based on the physical doses SD and TD was then recalculated into the corresponding EQD2 values.

Red columns indicate infringement of the dose constraints which is expected to be observed in the planning risk volume (PRV). The PRV contains the steep dose fall off between the dominant PTV (PTV_dom) and the organ at risk volume as depicted in Figure 1. Red columns were more frequently seen in PRVs and only occasionally in OAR volumes. 1= dose to OAR is dose to the organ at risk as segmented on the 3D planning CT. 2= constraints for biliodigestive anastomosis were identical with dose constraints for small bowel. 3= constraints employed for colon were defined for specific volumes (3 mL and 20 mL) but not for a maximal point dose.

Abbreviations:

# = number of fractions

BDA = biliodigestiven anastomosis

D_(x) = absolute dose to volume in mL

Dmax = maximum (point) dose

EqD2 α/β3 = equivalent dose in 2 Gy fractions at a specific α/β ratio [Gy] for late effects of OARs

n.a.; not available

OAR = organ at risk

PRV = planning risk volume

PTV = planning target volume

SD = single dose

SIP = simultaneous integrated boos

TD = total dose
