# Supplementary material for: Simultaneous integrated protection: A new concept for high-precision radiation therapy
Source: Strahlenther Onkol. 2016 Oct 18;192(12):886–94. doi: 10.1007/s00066-016-1057-x (PMC5122615; doi:10.1007/s00066-016-1057-x)
Supplement: Supplementary file 3 — Supplementary Table 2 Dosimetric specifics for the treatment plans shown in Figure 4. [file 66_2016_1057_MOESM3_ESM.docx]

Supplementary Table 2

| **SIP_OAR** | **PTV type** | **D_mean_** | **D_min_** | **D_max_** | **D_98_ Gy** | **D_95_ Gy** | **D_02_ Gy** | **V_95_ (%)** | **V_107_ (%)** |
| --- | --- | --- | --- | --- | --- | --- | --- | --- | --- |
| **Optic nerve**^&^  P=64.8 Gy  SD = 1.8 Gy | PTV_dom_  44.3 mL | 64.48 | 44.26 | 68.56 | 59.80 | 62.32 | 66.38 | 97.24 | 0.01 |
|  | PTV_SIP_  0.4 mL | 60.90 | 57.89 | 64.64 | 58.68 | 59.00 | 63.50 | 50.08 | 0.00 |
| **Brain-stem**  P=63 Gy  SD 1.8 Gy | PTV_dom_  59.1 mL | 63.01 | 50.54 | 66.96 | 56.85 | 58.63 | 65.89 | 84.10 | 0.00 |
|  | PTV_SIP_  2.5 mL | 55.10 | 52.19 | 59.16 | 52.83 | 53.10 | 58.00 | 0.00 | 0.00 |
|  | Brainstem  26.6 mL | 35.80 | 9.94 | 55.82 | 14.34 | 15.99 | 53.75 | 0.00 | 0.00 |
| **Hippo-campus** | PTV_dom_ | 35.00 | 18.01 | 40.44 | 30.31 | 32.18 | 38.35 | 87.38 | 6.09 |
|  | PTV_SIP_\Hippo | 21.08 | 9.10 | 36.57 | 10.96 | 11.86 | 33.94 | 3.27 | 0.00 |
|  | Hip bilateral | 11.01 | 8.70 | 20.24 | 8.93 | 9.06 | 15.48 | 0.00 | 0.00 |
|  | Hippoc., left | 11.17 | 8.70 | 20.24 | 8.89 | 9.03 | 15.91 | 0.00 | 0.00 |
|  | Hippoc., right | 10.77 | 8.80 | 17.16 | 8.99 | 9.09 | 14.36 | 0.00 | 0.00 |

\ sign for “minus” for Boolean operators, * minimum in skin / air regions; ^&^patient refuses orbital enucleation; P = prescribed dose
